# SAA1 increases NOX4/ROS production to promote LPS-induced inflammation in vascular smooth muscle cells through activating p38MAPK/NF-κB pathway

**DOI:** 10.1186/s12860-019-0197-0

**Published:** 2019-06-19

**Authors:** Mei-Hong Yu, Xi Li, Qian Li, Shi-Jing Mo, Yin Ni, Fang Han, Yi-Bin Wang, Yue-Xing Tu

**Affiliations:** 1 Department of ICU, Chunan First People’s Hospital, Hangzhou, 311700 China; 2 Centre of Laboratory Medicine, Zhejiang Provincial People’s Hospital, People’s Hospital of Hangzhou Medical College, Hangzhou, 310014 China; 3 Department of ICU, Zhejiang Provincial People’s Hospital, People’s Hospital of Hangzhou Medical College, Hangzhou, 310014 China; 4 Department of Cardiology, Chunan First People’s Hospital, Hangzhou, 311700 China

**Keywords:** Serum amyloid A1, Vascular smooth muscle cell, LPS, Inflammation, NOX-4/ROS, p38MAPK/NF-κB

## Abstract

**Background:**

To investigate the effects of serum amyloid A1 (SAA1) on lipopolysaccharide (LPS) -induced inflammation in vascular smooth muscle cells (VSMCs). SAA1 expression was detected in LPS induced VSMCs at different concentrations for different time by using Western blotting. After pre-incubation with recombinant SAA1 protein, VSMCs were treated with 1 μg/ml LPS for 24 h. The VSMCs were then divided into Control, SAA1 siRNA, Nox4 siRNA, LPS, LPS + SAA1 siRNA, LPS + Nox4 siRNA and LPS + SAA1 siRNA + Nox4 groups. MTT was performed to observe the toxicity of VSMCs. Lucigenin-enhanced chemiluminescence method was used to detect superoxide anion (O_2_^−^) production and NADPH oxidase activity. Quantitative real-time polymerase chain reaction (qRT-PCR) was used to determine expressions of inflammatory factors. Western blotting was used to determine expressions of NOX-4 and p38MAPK/NF-κB pathway related proteins.

**Results:**

LPS promoted SAA1 protein expression in a concentration−/time-dependent manner. Recombinant SAA1 protein could increase NOX4/ROS production and promote the release of inflammatory factors (*IL-1β*, *IL-6*, *IL-8*, *IL-17*, *TNF-α* and *MCP-1*) in LPS (1 μg/ml) - induced VSMCs. Besides, both SAA1 siRNA and NOX-4 siRNA could not only enhance the O_2_^−^ production and NADPH oxidase activity, but also up-regulate the protein expression of NOX4, the release of inflammatory factors, and the levels of p-p38 and p-NF-κB p65 in LPS-induced VSMCs. However, no significant differences in each index were observed between LPS group and LPS + SAA1 siRNA + Nox4 group.

**Conclusion:**

SAA1-mediated NOX4/ROS pathway could activate p38MAPK/NF-κB pathway, thereby contributing to the release of inflammatory factors in LPS-induced VSMCs.

## Background

According to the data issued by world health organization (WHO), cardiovascular disease-related death accounts for one third of all human clinical deaths globally, and almost 16 million people died of cardiovascular disease annually worldwide, among which 2.6 million were Chinese [1]. Recently, the incidence of cardiovascular disease is increased along with the rapidly uprising incidence of atheroselerosis, since atheroselerosis is the primary cause of cardiovascular disease, becoming a serious threat to human health [2]. As a chronic inflammatory disease of blood vessel wall, atheroselerosis is characterized with lipid accumulation and inflammatory cell aggregation in the blood vessel wall, as well as inflammatory reaction [3]. As a principal part of blood vessel wall, vascular smooth muscle cell (VSMC) will undergo phenotype switch once it is mechanically damaged, migrates to new intima, proliferates and secretes many inflammatory cytokines, and expresses inflammatory cell markers, thereby participating in chronic inflammatory response and atheroselerosis formation [4, 5]. Therefore, clarifying specific and molecular mechanisms of the inflammatory response of human VSMCs in atheroselerosis would provide new theoretical basis for prevention and treatment of atheroselerosis to some extent.

Serum amyloid A1 (SAA1), an acute phase protein, is widely accepted as an accurate and sensitive indicator of inflammation [6], which could be highly increased by 1000 times in vivo in the inflammatory state [7]. Recently, SAA1 was also found to be associated mostly with high density lipoproteins (HDL), involving in lipid metabolism in atherosclerosis [8, 9]. Additionally, Zhang et al. exhibited SAA-induced VSMC phenotypic modulation with decreased SMC marker and increased matrix synthesis-related marker [10], highlighting a close relation of SAA1 and atheroselerosis development. Besides, SAA1 was induced by lipopolysaccharide (LPS), which is associated with the pathological changes in several diseases [11], showing that SAA1 is associated with the expressions of LPS induced inflammatory factors. Nicotinamide adenine dinucleotide phosphate oxidase (NOX), a key enzyme to generate reactive oxygen species (ROS) in VSMCs, and other vascular endothelial cells [12, 13], exerts a crucial physiological function in redox signaling. And recent evidence supported that NOX4 activation and ROS production can participate in LPS-mediated inflammatory response [14]. Furthermore, Hatanaka et al. demonstrated that fibroblast proliferation and ROS production are stimulated by SAA upon chronic inflammatory conditions [15]. However, it is still unknown whether SAA1-mediated NOX4/ROS pathway involved in the release of LPS-induced inflammatory factors in VSMCs, as well as the specific mechanism.

Therefore, this study used the VSMCs isolated from rats in vitro to examine effects of SAA and LPS jointly interacting on VSMC on NOX4/ROS pathway. Finally, this study classified VSMCs into Control, SAA1 siRNA, Nox4 siRNA, LPS, LPS + SAA1 siRNA, LPS + Nox4 siRNA and LPS + SAA1 siRNA + Nox4 groups, to explore whether SAA1 siRNA could influence the release of LPS-induced inflammatory factor in VSMCs via mediating NOX4/ROS pathway, thereby providing a theoretical basis of the new target for the prevention and treatment of atheroselerosis.

## Results

### Effect of SAA1 on VSMCs viability

**Fig. 1:**
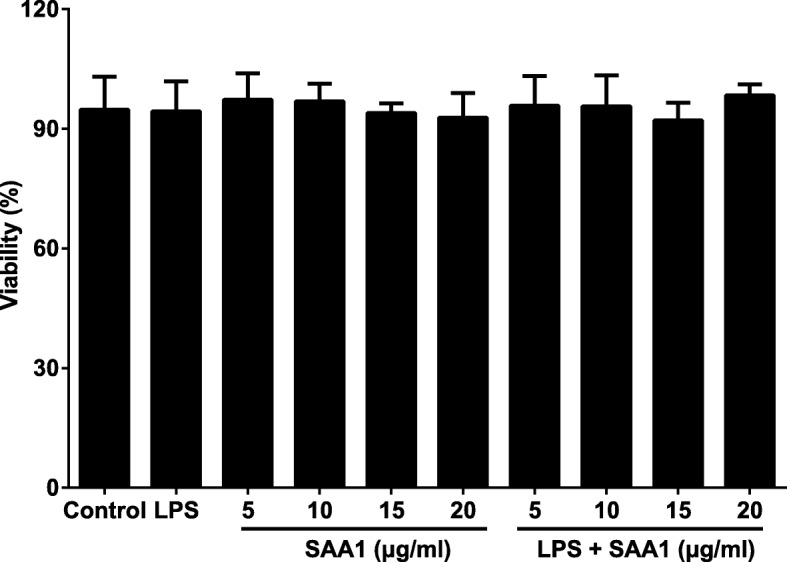
The toxicity of VSMCs was determined by the MTT assay (*n* = 3). Notes: The cells in control group received no treatment; to detect whether SAA1 or the combination of SAA1 with LPS was toxic to VSMCs, cells were treated with different concentrations of SAA1 (5, 10, 15, 20 μg/ml) with/without LPS (1 μg/ml) for 24 h

According to the previous study [16] and our result, LPS (1 μg/ml) and recombinant SAA1 protein (5, 10, 15, 20 μg/ml) did not show any toxicity in VSMCs. Besides, to detect whether SAA combined with LPS (1 μg/ml) was toxic to VSMCs, cells were treated with different concentrations of SAA1 (5, 10, 15, 20 μg/ml) and LPS (1 μg/ml) for 24 h, and were then subjected to the MTT assay to determine cell viability. The results indicated combination of SAA at concentrations between 0 and 20 μg/ml and LPS (1 μg/ml) did not affect VSMC viability (Fig. 1).

### Effects of LPS on SAA1 protein expression in VSMCs

**Fig. 2:**
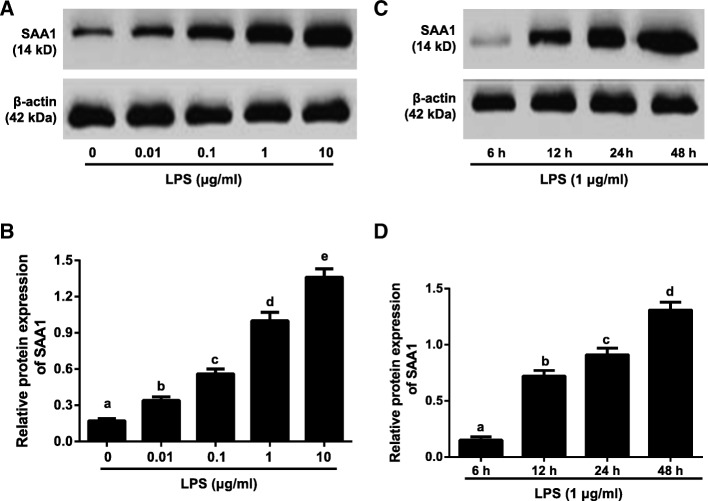
Effects of LPS on SAA1 protein expression in VSMCs detected by Western blotting (*n* = 3). Notes: **a**-**b**, The SAA1 protein expression in VSMCs induced by LPS with different concentrations (0, 0.01, 0.1, 1, 10 μg/ml) for 24 h; **c**-**d**, Detection of SAA1 protein expression in VSMCs induced by LPS (1 μg/ml) at different time points (6, 12, 24, 48 h). The same letters represented no significant differences, *P* > 0.05; and different letters indicated significant differences, *P* < 0.05

LPS (0, 0.01, 0.1, 1, 10 μg/ml) induced SAA1 protein expression was expressed in a concentration-dependent manner (Fig. 2a-b). Besides, SAA1 protein expression was increased in a time-dependent manner (6 h, 12 h, 24 h and 48 h) in VSMCs induced by LPS (1 μg/ml) (Fig. 2c-d).

### Effects of SAA1 on NOX4/ROS pathway in LPS-induced VSMCs

**Fig. 3:**
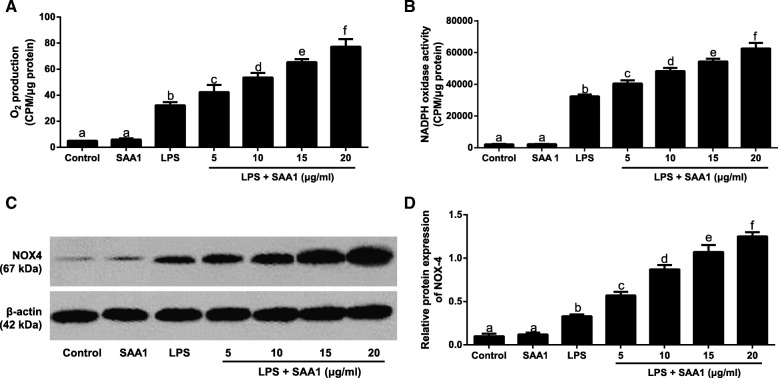
Effects of different concentrations of recombinant SAA1 protein on NOX4/ROS pathway in LPS-induced VSMCs (*n* = 3)**.** Notes: The levels of superoxide anion (O_2_^−^) production (**a**), NADPH oxidase activity (**b**), and NOX-4 protein expression (**c**-**d**) in VSMCs after pre-incubation with different concentrations of SAA (5, 10, 15, 20 μg/ml) for 24 h followed by the addition with 1 μg/ml LPS for 24 h; Some VSMCs were treated with SAA (20 μg/ml) alone for 24 h. The same letters represented no significant differences, *P* > 0.05; and different letters indicated significant differences, *P* < 0.05

**Fig. 4:**
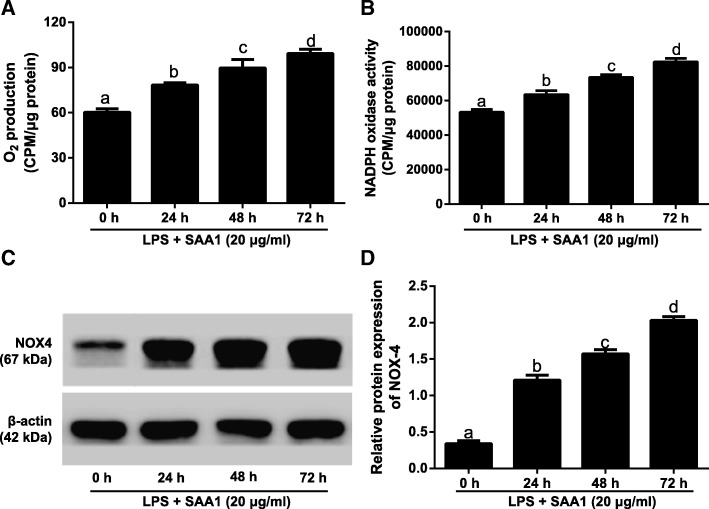
Effects of recombinant SAA1 protein treatment for different time points on NOX4/ROS pathway in LPS-induced VSMCs (*n* = 3). Notes: The levels of O_2_^−^ production (**a**), NADPH oxidase activity (**b**) and NOX-4 protein (**c**-**d**) in VSMC after pre-incubation with 20 μg/ml SAA1 for different time points (0, 24, 48, 72 h) followed by the addition with 1 μg/ml LPS for 24 h. The same letters represented no significant differences, *P* > 0.05; and different letters indicated significant differences, *P* < 0.05

To investigate the effects of SAA1 on NOX4/ROS pathway in LPS-induced VSMCs, after 24-h pre-incubation with recombinant SAA1 protein (5, 10, 15, 20 μg/ml), 1 μg/ml LPS was added to culture the cells for 24 h. As shown in Fig. 3, the increased levels of O_2_^−^, NADPH oxidase activity, NOX4 protein expression were presented in VSMCs in a dose-dependent manner. However, no significant differences of these indexes were found between control group and SAA1 group (all *P* > 0.05). In Fig. 4, pre-incubation with 20 μg/ml SAA1 could up-regulate the level of O_2_^−^, the activity of NADPH oxidase, and the expression of NOX-4 protein in LPS (1 μg/ml) - induced VSMCs in a time-dependent manner.

### Effects of SAA1 on LPS-induced expressions of inflammatory factors in VSMCs

**Fig. 5:**
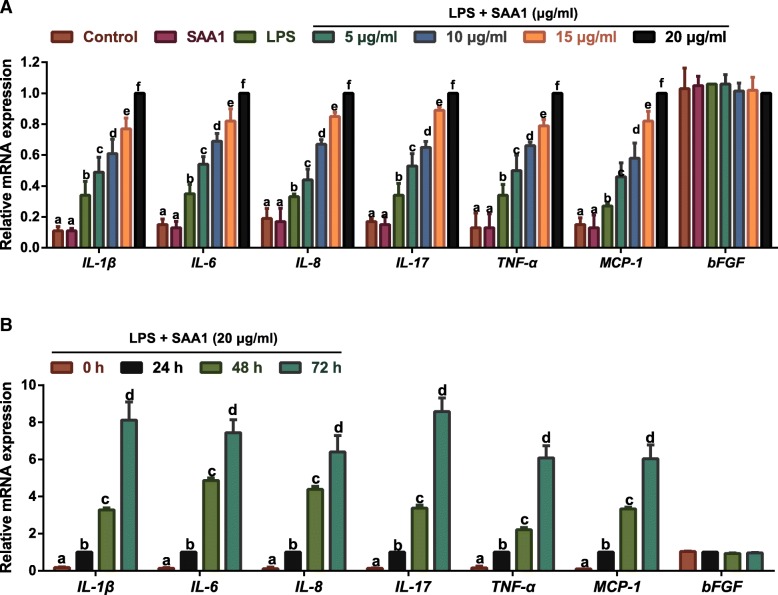
Effects of recombinant SAA1 protein on inflammatory factors in LPS-induced VSMCs detected by qRT-PCR (*n* = 3). Notes: **a**, The mRNA expressions of inflammatory factors (*IL-1β*, *IL-6*, *IL-8*, *IL-17*, *TNF-α*, *MCP-1* and *bFGF*) in VSMCs after pre-incubation with different concentrations of SAA (5, 10, 15, 20 μg/ml) for 24 h followed by the addition with 1 μg/ml LPS for 24 h; Some VSMCs were treated with SAA (20 μg/ml) alone for 24 h; **b**, The mRNA expressions of inflammatory factors in VSMCs (*IL-1β*, *IL-6*, *IL-8*, *IL-17*, *TNF-α*, *MCP-1* and *bFGF*) after pre-incubation with 20 μg/ml SAA for different time points (0, 24, 48, 72 h) and addition with 1 μg/ml LPS for 24 h. The same letters represented no significant differences, *P* > 0.05; and different letters indicated significant differences, *P* < 0.05

Considering the basic fibroblast growth factor (bFGF), an immunomodulatory factor during the early stages of inflammation [17], was not stimulated by LPS and SAA1 [18, 19], we determined its expression as negative control. The elevated expressions of inflammatory factors (including *IL-1β*, *IL-6*, *IL-8*, *IL-17*, *TNF-α* and *MCP-1*) were presented in LPS (1 μg/ml) -induced VSMCs treated with SAA1 in a dose- and time- dependent manner (Fig. 5).

### Effects of SAA1- mediated NOX4/ROS pathway on inflammatory factors in LPS-induced VSMCs

**Fig. 6:**
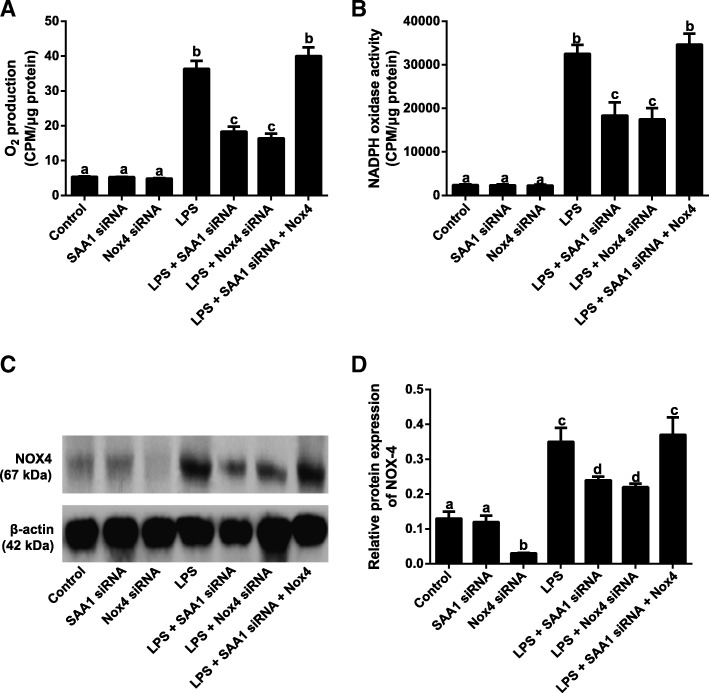
Inhibitory effect of SAA1 siRNA on the levels of O_2_^−^ production (**a**), NADPH oxidase activity (**b**) and NOX-4 protein (**c**-**d**) in LPS (1 μg/ml) -induced VSMCs via suppressing NOX4/ROS pathway (*n* = 3). Notes: The same letters represented no significant differences, *P* > 0.05; and different letters indicated significant differences, *P* < 0.05

**Fig. 7:**
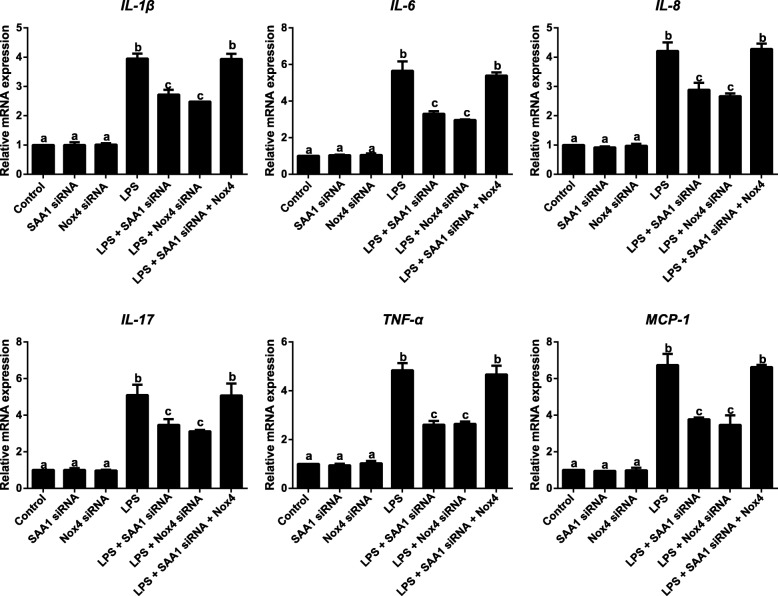
Inhibitory effect of SAA1 siRNA on inflammatory factors (*IL-1β*, *IL-6*, *IL-8*, *IL-17*, *TNF-α* and *MCP-1*) in LPS (1 μg/ml) -induced VSMCs via suppressing NOX4/ROS pathway detected by qRT-PCR (*n* = 3). Notes: The same letters represented no significant differences, *P* > 0.05; and different letters indicated significant differences, *P* < 0.05

Compared with Control group, the level of O_2_^−^, the activity of NADPH oxidase and the protein expression of NOX4 were increased in LPS-induced VMSCs, which were much lower in LPS + SAA1 siRNA and LPS + Nox4 siRNA groups than LPS group (all *P* < 0.05). However, no significant differences presented between LPS group and LPS + SAA1 siRNA + Nox4 group (all *P* > 0.05, Fig. 6). According to the qRT-PCR results demonstrated in Fig. 7, LPS could increase the expressions of *IL-1β*, *IL-6*, *IL-8*, *IL-17*, *TNF-α* and *MCP-1* in VSMCs. In comparison with LPS group, LPS + SAA1 siRNA and LPS + Nox4 siRNA groups presented a significant reduction in expressions of inflammatory factors in VSMCs (*P* < 0.05), but showed no differences in comparison with LPS + SAA1 siRNA + Nox4 group (all *P* > 0.05).

### Effects of SAA1 mediated-NOX4/ROS on p38MAPK/NF-κB pathway in LPS-induced VSMCs

**Fig. 8:**
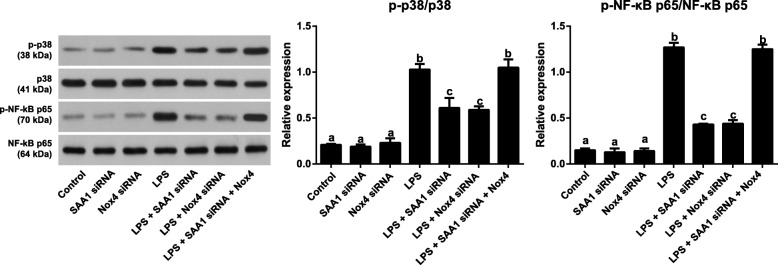
Inhibitory effect of SAA1 siRNA on p38MAPK/NF-κB signaling pathway via suppressing NOX4/ROS pathway in LPS (1 μg/ml) -induced VSMCs (*n* = 3). Notes: The same letters represented no significant differences, *P* > 0.05; and different letters indicated significant differences, *P* < 0.05

Seen from Fig. 8, evident enhancements in the levels of p-p38 and p-NF-κB p65 were found in LPS-induced VSMCs (all *P* < 0.05). LPS + SAA1 siRNA and LPS + Nox4 siRNA groups presented lower levels of p-p38 and p-NF-κB p65 than LPS induction alone (all *P* < 0.05), but there was no significant differences between LPS + SAA1 siRNA + Nox4 group and LPS group (all *P* > 0.05). Moreover, no statistical differences were found in the levels of p38 and NF-κB p65 among each group (all *P* > 0.05).

## Discussion

Atheroselerosis is not only a common chronic disease that endangers human health but also a vital cause of various cardiovascular diseases, and its pathogenesis is still not completely elucidated because of its extremely complicated etiology, hidden onset and slow course of disease [20]. Generally, atheroselerosis is a complex and multifactorial process of chronic inflammation with basic pathological changes of inflammation, like degeneration, exudation and proliferation, in which inflammation response plays a crucial role from beginning to the end of vascular stenosis, as indicated by Ross et al. in 1999 [21]. Therefore, it is of great significance to better understand the pathogenesis of inflammation response in atheroselerosis.

As shown by our results, LPS could promote SAA1 protein expression in VSMCs in a concentration- and time-dependent manner, which was in accordance with results of the study by Nakarai et al. in which several times higher of SAA1 in adipocytes was found with LPS stimulation for 4, 8, 12 and 24 h [22]. To our knowledge, LPS, the main component on the outer membrane of gram negative bacteria, could stimulate various types cells, including macrophages, endothelial cells and VSMCs, and induce a series of pro-inflammatory genes (TNF-α, IL-17, IL-1β, IL-6, and IL-8) and proteins like CRP and SAA in acute phase response, which is the main cause of inflammation [23, 24]. Besides, the above pro-inflammatory factors were also interacted with SAA1 in cardiovascular disease, including atherosclerosis [9, 25]. Of note, LPS, even at low levels, was reported to exert great effects on the development of atheroselerosis [26], and circulating levels of SAA1 shared a strong correlation with the extent of atheroselerosis in the aorta [27], indicating that LPS may be involved in the occurrence and development of atheroselerosis through affecting the expression of SAA1, but the specific mechanism is not yet clear.

Furthermore, our findings demonstrated that LPS up-regulated the production of O_2_^−^, the activity of NADPH oxidase, the protein expression of NOX4, and the release of inflammatory factors in VSMCs. ROS is the generic terms of active oxygen contained compounds, such as O_2_^−^, hydrogen peroxide (H_2_O_2_), and hydroxyl radical (OH) [28], whose production is closely correlated with the atheroselerosis progression [29]. Under normal physiological condition, vascular wall can produce ROS at low level, but when the balance of ROS production and clearance was lost, the excessive ROS can damage the vascular wall [30]. In recent years, the activation of NOX in atherosclerotic plaque has been found to be able to mediate ROS production and then induce oxidative stress so as to enhance the proliferation and migration of VSMCs and become a vital source of various inflammatory factors, thereby contributing to the occurrence and development of atheroselerosis [31, 32]. As we know, NOXs family consists of seven members including Nox1, Nox2 (gp91phox), Nox3, Nox4, Nox5, bifunctional oxidase Duox1 and Duox2 [33], among which Nox4 had a critical role in maintaining the functional status of VSMCs. For example, Lassègue et al. reported the NOX-4 expression was dramtically enhanced in the process of VSMC differentiation after vascular wall injury [34]. Moreover, Poldip2, a novel regulator of Nox4, could stimulate the enzymatic activity of NOX-4, consequently mediating focal adhesion turnover and VSMC migration, as reported by Lyle et al. [35]. Meanwhile, ROS production was evidently reduced, as well as expression of inflammatory factors, like IL-8 and MCP-1, after inhibition of NOX-4 [36]. In this study, the NOX4/ROS pathway was further activated in LPS-induced VSMCs with the SAA treatment, and the release of inflammatory factors was up-regulated in a concentration- and time-dependent manner, which was in accordance with previous studies [15, 37]. There was evidence that acute-phase SAA1 was found to be related to the presence of oxidative stress in atheroselerosis [38], and in turn NOX4/ROS-induced oxidative stress was believed to act as a functional role in the pathogenesis and progression of disease, including atheroselerosis [39], which provides the possibility that SAA may be responsible for the formation of atheroselerosis through affecting the expressions of inflammatory factors via mediating the NOX4/ROS signaling pathway.

In addition, the MAPK signaling pathway is also important in the inflammatory process [40]. P38MAPK, as the most important member in the MAPK family to control inflammatory response, was often phosphorylated and activated by inflammatory mediators, cytokines and LPS [41, 42], and meanwhile, it could stimulates the nuclear transcription factor NF-κB (p65) to jointly facilitate inflammatory response, which is one of the most significant approaches to induce atheroselerosis [43]. At present, many studies have found SAA was able to regulate the gene transcription of various inflammatory mediators via mediating the p38MAPK/NF-κB pathway, for instance, the study of Chung et al. demonstrated that SAA1 activated NF-κB and MAPK pathway to induce cytokines [44]. As revealed by He and the team, SAA1 was identified to activate the transcription factors of NF-κB and p38MAPK in stimulated THP-1 cells [37]. More importantly, Sildenafil and Curcumin respectively exert anti-inflammatory effects on LPS-induced neuroglia (N9) cells and VSMCs through suppressing the MAPK/NF-κB pathway, partial because of the inhibition of NADPH-mediated intracellular ROS production [45, 46]. Consistently, our findings showed that Inhibition of SAA1 suppressed the NOX4/ROS pathway in LPS induced VSMCs to decrease the levels of p-p38 and p-NF-κB (p65), and to inhibit the release of inflammatory factors, which was reversed by the treatment of Nox4 lentiviral activation particles, implying that SAA-mediated NOX4/ROS pathway can stimulate the p38MAPK/NF-κB pathway to promote the release of inflammatory factors in LPS-induced VSMCs.

## Conclusions

To sum up, our findings presented that LPS can promote SAA1 protein expression in a concentration−/time- dependent manner. Additionally, inhibition of SAA1 may suppress the NOX/ROS pathway via mediating the p38MAPK/NF-κB pathway, thereby improving the release of inflammatory factors in LPS-induced VSMCs.

## Methods

### Experimental animals

The experimental animals were healthy adult male Sprague-Dawley (SD) rats (Shanghai SLAC Laboratory Animal Co., Ltd., Shanghai, China) weighting 150–200 g, and then were fed with free food and water, kept in a clean animal room at 21–23 °C in humidity of 60% ± 5% and normal circadian rhythm. The animal experiment design got the permission from the experimental animal ethics committee of our hospital, and all the research behaviors for the experimental animals strictly complied with the relevant animal protection and use regulations issued by International Association for the Study of Pain [47].

### VSMCs culture

The rats were executed by cervical dislocation to take out the thoracic aorta under aseptic surgical conditions and put in sterile phosphate buffer solution (PBS). Next, the blood vessel was cut longitudinally and removed the endothelium and adventitia to get the smooth muscle tissues, which were cut into pieces of 1 mm^2^ and incubated at 37 °C with CO_2_. DMEM containing 20% fetal bovine serum (FBS) was added for passages, after the cells covered the bottom of the bottle. The cells used in this study were between passages 5 and 12. Subsequently, cells were digested by 0.05% trypsin and added DMEM with 10% FBS, followed by a centrifugation at 3500 rpm for 7 min. Later, the cells were resuspended using DMEM with 10% FBS. The purity of the VSMCs was determined by the positive staining of SM a-actin, the positive cells are over 95%.

### MTT assay

To observe the toxicity of VSMCs after treatments with recombinant SAA1 protein (0, 5, 10, 15, 20 μg/ml) or the combination of recombinant SAA1 protein (0, 5, 10, 15, 20 μg/ml) and LPS (1 μg/ml), cells were seeded in 96-well plates at a density of 4000 cell/well to determine cell viability by the MTT assay. After VSMCs were incubated with 5 mg/mL MTT at 37 °C for 4 h, the dark blue formazan crystals were solubilized with DMSO for 15 min. The absorbance at 490 nm was measured by using a microplate reader. We purchased recombinant SAA1 protein from ProSci incorporated (Poway, CA, USA). LPS from *Escherichia coli* 0111:B4 was purchased from Sigma Chemical Co (St Louis, MO, USA).

### Cell grouping

VSMC were classified into Control group (no treatment), SAA1 siRNA group (VSMCs were transfected with SAA1 siRNA for 48 h), Nox4 siRNA group (VSMCs were transfected with Nox4 siRNA for 48 h), LPS group (VSMCs were exposed to 1 μg/ml LPS for 24 h), LPS + SAA1 siRNA group (VSMCs were transfected with SAA1 siRNA for 48 h followed by exposed to 1 μg/ml LPS for 24 h), LPS + Nox4 siRNA group (VSMCs were transfected with Nox4 siRNA for 48 h followed by exposed to 1 μg/ml LPS for 24 h), and LPS + SAA1 siRNA + Nox4 group (VSMCs were transfected with SAA1 siRNA and Nox4 lentiviral activation particles for 48 h followed by exposed to 1 μg/ml LPS for 24 h). SAA1 siRNA, Nox4 siRNA and Nox4 lentiviral activation particles were obtained from Santa Cruz Biotechnology (Santa Cruz, CA, USA).

### Quantitative real-time polymerase chain reaction (qRT-PCR)

The Trizol method (Takara Biotechnology Ltd., Dalian, China) was used to extract the total RNA, which was detected for the OD260/280 value by ultraviolet spectrometry and stored at − 80 °C. On the basis of gene sequence published in the Genbank database, primers were designed with Primer 5.0 software and synthesized by Shanghai Biological Engineering Co., Ltd. (Shanghai, China). The reverse transcriptase PCR of total RNA was carried out following the procedures provided by cDNA transcript kit (Thermo Fisher Inc., Waltham, MA, USA). Following the procedures of SYBR Green PCR Master Mix kit (Takara, Japan), PCR amplification reaction conditions consisted of 95 °C for 10 min and 40 cycles of 95 °C for 15 s and 60 °C for 1 min. Taking GAPDH as internal reference gene, the relative expression level of target gene was calculated using 2^-△△^Ct. Each experiment was repeated in triple duplicate.

### Western blotting

The cell protein was extracted to determine its concentration according to instructions of BCA protein assay kit (Pierce, Thermo Fisher Scientific, USA), followed by adding loading buffer for 10 min boiling at 95 °C. After loaded into 10% sodium dodecyl sulfate-polyacrylamide gel electrophoresis (SDS-PAGE), the total protein of 60 μg was transferred to polyvinylidene fluoride (PVDF) membrane and sealed with 5% bovine serum albumin (BSA) at room temperature for 1 h. Next, PVDF membrane was incubated with anti-SAA1 (ab171030, 1 μg/ml), anti-NADPH oxidase 4 (ab133303, 1/2000), anti-phospho-p38 (ab4822, 1/1000), anti-p38 (ab197348, 1/500), anti-phospho-NF-kB p65 (ab86299, 0.04 μg/ml), anti-NF-kB p65 (ab16502, 1 μg/ml) and β-actin (ab227387, 1/20000) (all purchased from Abcam, Cambridge, MA, USA) at 4 °C overnight. With tris buffered saline with Tween (TBST) washing 3 times/5 min, PVDF membrane was incubated with goat anti-rabbit IgG H&L (HRP) (ab6721, 1/5000, Abcam, Cambridge, MA, USA) for 1 h and washed 3 times/5 min. Taking β-actin as loading control, PVDF membrane was developed by chemiluminescent reagent using Bio-rad Gel Dol EZ imager (GEL DOC EZ IMAGER, Bio-rad, California, USA). The gray value of target band was analyzed using Image J software. The experiment was repeatedly carried out in triple.

### Determination of NADPH oxidase activity and superoxide anion (O_2_^−^) production

VSMCs were lysed followed by the centrifugation at 29,000 g for 30 min at 4 °C. The pellets were resuspended in the lysis buffer and designated the membrane fraction. After washed by oxygenated Kreb-Hepes buffers, the membrane extract (40 μg) were scintillation vials supplemented with lucigenin (5 μM). We used a liquid scintillation counter (Wallac 1409; Perkin Elmer Life Science, St Laurent, Quebec, Canada) to scale the emitted luminescence for 5 min. With evaluation of the average luminescence value, the concentration of proteins in each sample was used to classify the background value subtracted and the result. Then, we determined the emitted luminescence for basal superoxide anion production. With addition of 0.1 mM NADPH, a liquid scintillation counter (Wallac 1409; PerkinElmer Life Science) was used for a continuous measurement of luminescence for 5 min. The NADH induced luminescence value minus the basal superoxide-induced luminescence was the NADPH oxidase activity. We repeated each experiment for three times.

### Statistical analysis

The collected data was analyzed using SPSS 21.0 software (SPSS, Inc., Chicago, IL, USA). All values were represented as mean ± standard deviation ($\overline{x}$ ± s), which were analyzed by Mann-Whitney rank sum test and Kruskal-Wallis test. All differences were believed significant at *P* < 0.05.

## Abbreviations

BSA: bovine serum albumin
H_2_O_2_: hydrogen peroxide
LPS: lipopolysaccharide
NOX: Nicotinamide adenine dinucleotide phosphate oxidase
O_2_^−^: superoxide anion
PVDF: polyvinylidene fluoride
qRT-PCR: Quantitative real-time polymerase chain reaction
ROS: oxygen species
SAA1: serum amyloid A1
SDS-PAGE: sodium dodecyl sulfate-polyacrylamide gel electrophoresis
VSMCs: vascular smooth muscle cells
